# Anthropometry-based 24-h urinary creatinine excretion reference for Chinese children

**DOI:** 10.1371/journal.pone.0197672

**Published:** 2018-05-23

**Authors:** Wei Wang, Cong Du, Laixiang Lin, Wen Chen, Long Tan, Jun Shen, Elizabeth N. Pearce, Yixin Zhang, Min Gao, Jianchao Bian, Xiaoming Wang, Wanqi Zhang

**Affiliations:** 1 The Department of Nutrition and Food Science, School of Public Health, Tianjin Medical University, Tianjin, China; 2 Tianjin Institution of Endocrinology, Tianjin Medical University, Tianjin, China; 3 Department of Sanitary Chemistry, Tianjin Medical University, Tianjin, China; 4 Section of Endocrinology, Diabetes and Nutrition, Boston University School of Medicine, Boston, MA, United States; 5 The Department of Iodine Deficiency Disorders, Shandong Institute for Endemic Disease Control and Research, Shangdong Province, China; The University of Western Ontario, CANADA

## Abstract

To establish 24-h urinary creatinine excretion reference ranges based on anthropometry in healthy Chinese children, a cross-sectional survey was conducted using twice-sampled 24-h urine and anthropometric variables. Age- and sex-specific 24-h creatinine excretion reference ranges (crude and related to individual anthropometric variables) were derived. During October 2013 and May 2014, urine samples were collected. Anthropometric variables were measured in the first survey. Data of 710 children (377 boys and 333 girls) aged 8–13 years who completed the study were analyzed. No significant difference was observed in 24-h urine volumes between the two samples (median [interquartile range): 855.0 [600.0–1272.0) mL vs. 900.0 [660.0–1220.0) mL, P = 0.277). The mean 24-h urine creatinine excretion was regarded as representative of absolute daily creatinine excretion in children. Sex-specific, body-weight-adjusted creatinine excretion reference values were 15.3 mg/kg/day (0.1353 mmol/kg/day) for boys and 14.3 mg/kg/day (0.1264 mmol/kg/day) for girls. Differences were significant between boys and girls within the same age group but not across different age groups within the same sex. Ideal 24-h creatinine excretion values for height were derived for potential determination of the creatinine height index. These data can serve as reference ranges to calculate ratios of analyte to creatinine. The creatinine height index can be used to assess somatic protein status.

## Introduction

Creatine is present in the muscle as phosphocreatine. Creatinine is formed by the nonenzymatic cleavage of phosphocreatine. Approximately 2% of the body’s creatine stores are converted into creatinine daily [1]. Once creatinine is formed, it diffuses from the cells and ultimately appears in the urine after glomerular filtration and, to a small extent, tubular secretion [2]. Because the amount of creatinine excretion reflects the body’s muscle mass, measurement of urinary creatinine excretion may be used to assess lean body mass or body composition [3]. In addition, creatinine is excreted in urine at a fairly constant rate throughout the day; therefore, ratios of an analyte to creatinine are used to estimate the analyte’s excretion rates [4–9]. Some analytes determined in spot urine samples are routinely normalized to urinary creatinine because although there may be considerable intraindividual variation in values during the day, especially after ingestion of liquids or sweating, creatinine is excreted at a relatively constant rate throughout the day [10–14]. Certain analytes’ 24-h excretion rates in urine can be calculated by multiplying the ratio of the analyte by the urinary creatinine using age- and sex-specific creatinine reference values. Therefore, as a reference comparator for analyses performed on spot urine and timed urine samples, accurate reference values for 24-h urinary creatinine excretion are essential.

Because the reference range for urinary creatinine varies considerably due to anthropometric factors including weight, height, and even ethnicity, the results of studies from other population groups should not be applied to other populations. There is a lack of large-scale epidemiological studies on 24-h urinary creatinine excretion in Chinese children [15, 16]. The present study aimed to establish anthropometry-based age- and sex-specific reference values for the urinary 24-h creatinine excretion for 8–13-year-old Chinese children based on a large population-based sample.

## Methods

### Subjects

A total of 981 healthy children involved in this study were selected by "one-stage" cluster sampling plan. Three elementary schools were randomly selected from Dezhou, China. All of the children studying in each selected elementary school were sampled. The regions in which three selected elementary schools are situated have similar climate, economic structure, culture, and dietary habits. All subjects underwent a physical evaluation and biochemical testing of blood to ensure they were healthy children without chronic diseases. Each child was asked to fill out a 3-day dietary record, and energy and protein intakes were estimated.

### Urine sample collections

Two 24-h urine samples were obtained from each participant within 1 month (28–30 days). The first and second samples were collected in October and November 2013, respectively, in Ningjin County and in April and May 2014, respectively, in Lingxian County. Children were required to void their bladders in the morning before initiating the 24-h urine sample collection. From then on, all the urine passed for the next 24 h, including the first morning void of the next day, was collected in polyethylene bottles. Teachers and parents were trained to assist children in completing the study. After the last micturition, no one was allowed to open the container again. Children were instructed to inform teachers or investigators if they forgot or spilled urine samples, and in such cases, they were asked to repeat the collection. Investigators asked children about missed urine samples. Only 24-h samples with no reported missed void were considered acceptable. Urine volumes were measured, and after mixing thoroughly, 5-mL urine from each 24-h sample was removed as the test sample. All urine samples were stored at ≤4°C before delivery to the research institute and at ≤20°C until testing.

### Determination of urinary creatinine

Urinary creatinine was assessed at the Key Laboratory of Hormone and Development (Ministry of Health), Metabolic Diseases Hospital and Tianjin Institute of Endocrinology, Tianjin Medical University. It was measured using a national standard method (Jaffe method[17]) with a spectrophotometer (WFJ7200, Unico (Shanghai) Instrument Co., Ltd.). Two samples of certified reference material (lot no. 66731, 66732; Bio-Rad Laboratories, Inc.) with mean certified creatinine concentrations of 0.649 g/L (95% reference range: 0.489, 0.808 g/L) and 1.469 g/L (95% reference range: 1.050, 1.887 g/L), respectively, were run with each batch of samples. Urinary creatinine was multiplied by urine volume to determine the 24-h urinary creatinine excretion. The within-run coefficient of variation (CV), between-day CV, and total CV imprecision of urinary creatinine was 1.1%, 2.4%, and 3.2%, respectively.

### Statistical analysis

Data are reported as means ± standard deviation (SD) for intake of energy and protein, body height, body weight, and BMI. The values of 24-h urinary creatinine excretion are reported as the mean ± SD or median and 95% confidence intervals (CI) as required. The Kolmogorov–Smirnov (KS) test was performed to assess for normality of distribution of variables.

Wilcoxon signed rank tests were used to determine the difference between medians of urine volumes for the first and second 24-h samples. Student’s t-test was performed to detect the difference between means of 24-h urinary creatinine excretion for boys and girls. One-way ANOVA was used to test for differences of 24-h urinary creatinine excretion related to body weight among age groups for boys and girl separately. Pearson’s correlation coefficients were used to determine correlations between 24-h creatinine excretion and anthropometric characteristics. Multiple linear regression analysis was performed to further clarify the relationships between 24-h creatinine excretion and sex, age, and body height and weight. Cases containing any missing data were not were included in the analysis. The ideal 24-h urinary creatinine excretion values for height were calculated by line interpolation. The molecular weight of creatinine is 113.1 g/mol. Thus, mg/kg/day can be transformed into mmol/kg/day by dividing it by 131.1. All statistical analyses were performed using the Statistical Package for Social Sciences version 22.0 (IBM SPSS Inc.), and Graph Prism 6.0c (Graph Pad Software Inc.). Significance was set at two-tailed α<0.05.

### Ethics statement

This study was conducted according to the guidelines laid down in the Declaration of Helsinki, and all procedures involving human subjects were approved by the Medical Ethics Committee of Tianjin Medical University. Written informed consent was obtained from all parents or caretakers of participating children before the study.

## Results

### Baseline data

**Fig 1:**
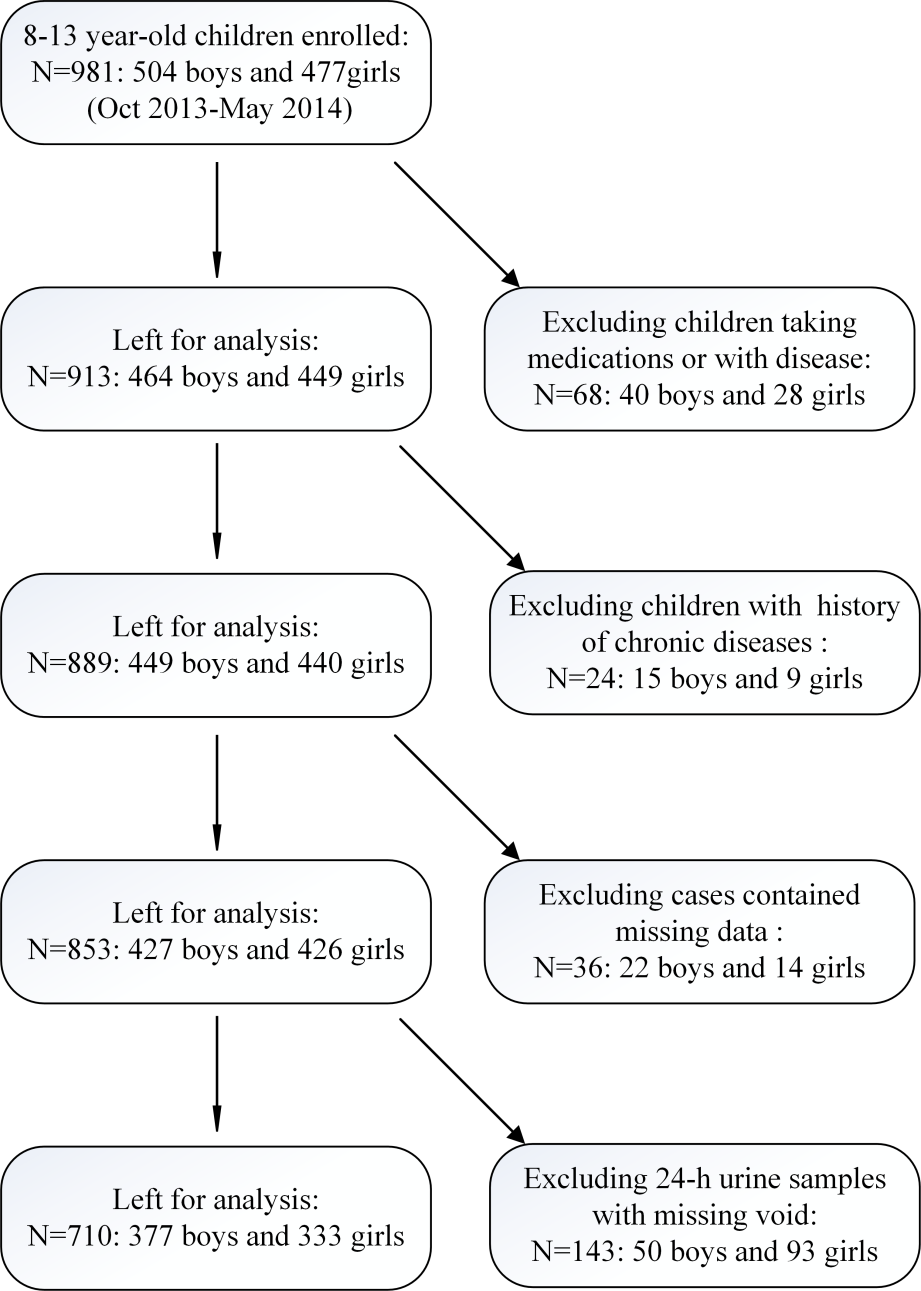
Flowchart of the study population.

A total of 710 children (377 boys and 333 girls) completed the study and met the inclusion criteria (Fig 1). Anthropometric characteristics and energy and protein intakes are shown in Table 1. As expected, both height and weight increased significantly with age (P < 0.001).

**Table 1:** Baseline anthropometric characteristics according to age and sex.

Sex and age group (yr)	Energy (kcal)		Protein (g/d)		Body height (cm)		Body weight (kg)		BMI (kg/m^2^)	
	Mean	SD	Mean	SD	Mean	SD	Mean	SD	Mean	SD
Boys										
8 (n = 9)	1681.2	592.1	73.4	21.1	132.3	2.7	28.5	2.4	16.3	1.3
9 (n = 65)	1593.8	448.2	63.3	16.1	136.1	5.4	30.9	4.8	16.6	2.0
10 (n = 112)	1924.0	611.9	78.9	28.1	141.9	5.7	35.1	6.4	17.4	2.5
11 (n = 95)	2017.8	520.3	79.7	16.8	145.5	6.8	38.4	7.6	18.0	2.8
12 (n = 64)	2067.7	741.2	81.9	26.1	150.5	6.7	42.8	9.8	18.7	3.2
13 (n = 32)	2166.8	966.4	84.2	32.0	154.7	6.6	44.5	8.4	18.5	2.5
Girls										
8 (n = 21)	2014.5	1080.9	74.6	33.4	129.7	6.9	29.3	5.1	17.4	2.6
9 (n = 58)	1769.1	819.5	70.1	27.0	136.4	5.4	30.6	4.6	16.4	1.9
10 (n = 101)	1949.3	691.9	75.0	22.8	140.4	7.7	33.6	6.9	16.9	2.6
11 (n = 92)	1924.7	617.5	72.9	18.1	146.6	7.5	37.0	8.1	17.1	2.5
12 (n = 45)	1794.1	406.9	69.5	15.0	152.0	7.3	41.2	7.7	17.7	2.5
13 (n = 16)	1928.3	405.5	73.6	24.1	156.7	7.1	47.1	10.6	19.0	3.4

### Urine creatinine excretion in children and associations with anthropometric factors

The medians of 24-h urine volumes for the two samples were 855.0 (600.0–1272.0) mL and 900.0 (660.0–1220.0) mL, respectively. And the difference was not significant between the two samples (P = 0.277). The mean values of 24-h creatinine excretion for the two samples were 522.4 ± 206.8 and 60.37 ± 211.0 mg/day for boys, and were 448.9 ± 168.9 and 561.5 ± 179.0 mg/day for girls.

The mean 24-h creatinine excretion of both collections was calculated and was regarded as being representative of absolute daily creatinine excretion for each individual. The unadjusted mean 24-h creatinine excretion values for boys and girls were 565.0 ± 171.5 and 502.0 ± 141.5 mg/day, respectively (P < 0.001).

**Fig 2:**
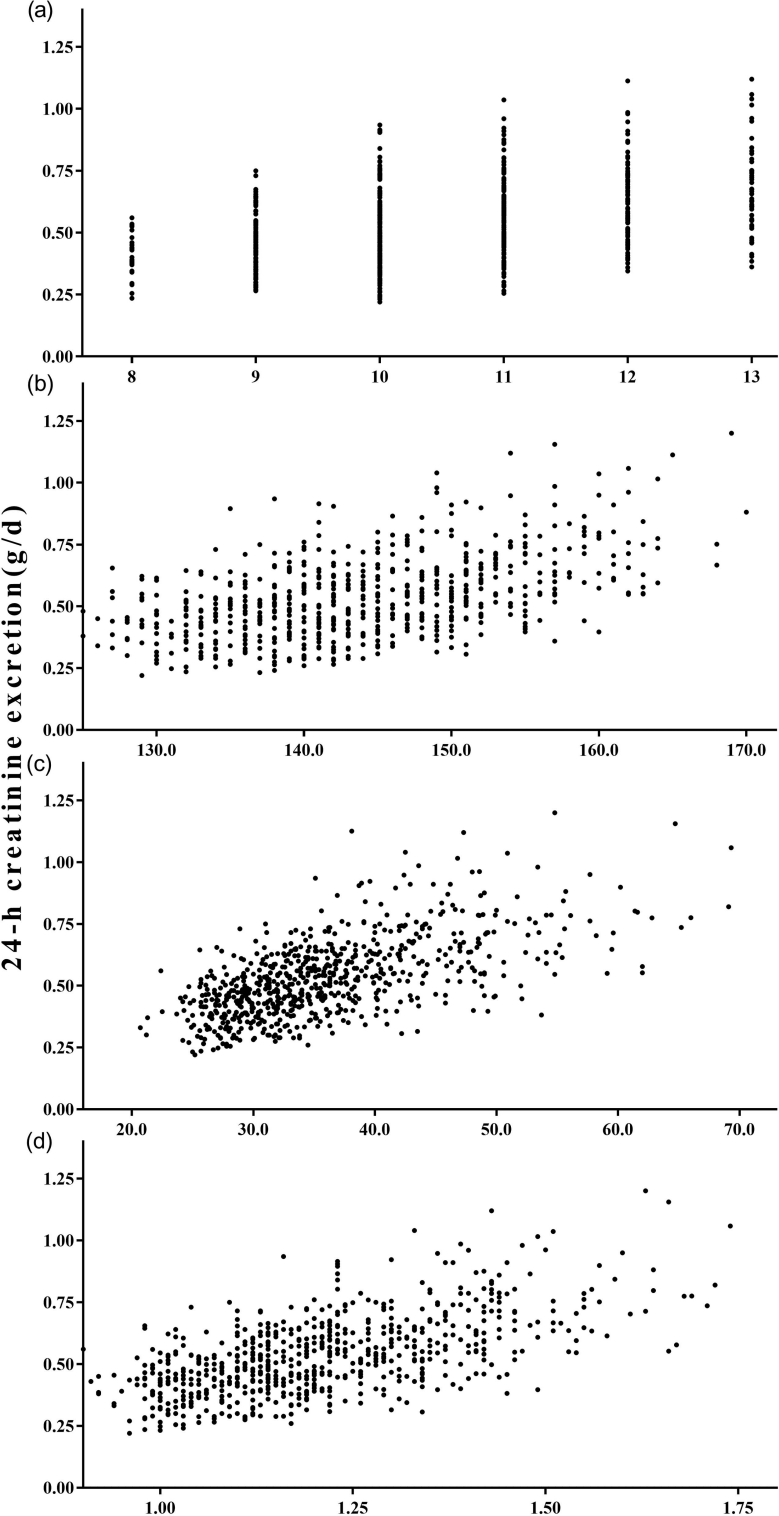
24-h urinary creatinine excretion in children. (a) age-related excretion rates (r = 0.4291, P<0.001); (b) body-height-related excretion rates (r = 0.5141, P<0.001); (c) body-weight-related excretion rates (r = 0.6278, P<0.001); (d) body-surface-area-related excretion rates (r = 0.6255, P<0.001).

The mean 24-h creatinine excretion in children significantly correlated with age (r = 0.4291, P < 0.001), body height (r = 0.5141, P < 0.001), body weight (r = 0.6278, P < 0.001), and BSA (r = 0.6255, P < 0.001) (Fig 2). Multiple linear stepwise regression analysis was applied to examine the independent effect of each anthropometric factor on children’s absolute daily creatinine excretion. After adjustment for sex, body height, and body weight, it was found that urinary creatinine excretion in children significantly increased with age (unstandardized β = 0.017, P < 0.001) and body weight (unstandardized β = 0.010, P < 0.001), and it differed between boys and girls (unstandardized β = 0.041, P < 0.001).

### 24-h urinary creatinine excretion reference values for Chinese children according to anthropometric factors

Because 24-h creatinine excretion was much more closely associated with body weight than with age, body height, or BSA, the individual ratios of creatinine to body weight were calculated to control for body-composition changes during growth. The corresponding sex-specific reference values for body weight-related 24-h urinary creatinine excretion for 8–13-year-old children are shown in Table 2. The differences between boys and girls within the same age group were significant, but values did not differ across age groups within the same sex. Thus, the mean value for body-weight-related 24-h urinary creatinine excretion for 8–13-year-old boys was 15.3 ± 3.6 mg/kg/day (equivalent 0.1353 ± 0.03183 mmol/kg/day), and that for 8–13-year-old girls was 14.3 ± 3.2 mg/kg/day (equivalent 0.1264 ± 0.02829 mmol/kg/day).

**Table 2:** 24-h urinary creatinine excretion related to body weight in 8–13 y healthy children.

	Creatinine excretion (mg/kg/d) *						
	8	9	10	11	12	13	Total^#^
Boys							
Value ^a^^b^	15.1	15.4	15.2	15.4	15.4	15.2	15.3
SD	3.4	3.3	3.7	3.6	4.1	3.6	3.6
Median ^c^	16.3	15.4	15.3	15.3	15.2	14.3	15.1
5^th^ percentile	9.1	10.3	8.7	9.3	9.5	11.2	9.5
95^th^ percentile	17.5	20.8	21.1	22.0	22.6	24.0	21.7
Girls							
Value ^a^^d^	14.2	14.2	14.2	14.5	14.5	14.4	14.3
SD	2.7	3.6	3.5	3.0	2.6	2.4	3.2
Median ^e^	14.2	13.6	14.0	14.5	14.4	13.7	14.2
5^th^ percentile	9.3	9.3	8.6	9.6	10.2	10.9	9.4
95^th^ percentile	18.6	21.2	20.4	20.0	19.1	19.3	19.9

* Values in mg/kg/day can be transformed into mmol/kg/day by dividing them by 113.1.

^#^ t = 3.890, P<0.001

^a^ n values given in Table 1.

^b^ F = 0.033, P = 0.999

^c^ χ^2^ = 0.613, P = 0.987

^d^ F = 0.141, P = 0.983

^e^χ^2^ = 1.495, P = 0.914

Using body height as a potential determinant, 24-h urinary creatinine excretion according to defined height groups are given in Table 3. Ideal 24-h urinary creatinine for height was also calculated (Table 4).

**Table 3:** 24-h urinary creatinine excretion stratified by sex and height.

Height group	Height ^a^	Body Weight(kg)		Creatinine (mg/d)	
(cm)	(cm)	Mean	SD	Mean	95%CI
Boys (n = 377) ^b^					
125.0–129.9 (n = 16)	128.2	26.9	2.4	458.6	392.0, 525.2
130.0–134.9 (n = 35)	132.7	28.7	2.6	459.1	419.1, 499.2
135.0–139.9 (n = 70)	137.7	31.9	4.4	511.7	479.9, 543.5
140.0–144.9 (n = 86)	142.2	35.1	4.9	526.6	493.6, 559.6
145.0–149.9 (n = 77)	147.2	38.8	6.3	580.7	546.9, 614.5
150.0–154.9 (n = 52)	151.9	43.4	6.1	629.0	585.6, 627.3
155.0–159.9 (n = 26)	157.0	47.7	6.5	708.5	644.6, 772.5
160.0–164.9 (n = 11)	162.2	53.9	8.0	799.9	652.0, 947.9
165.0–169.0 (n = 4)	167.8	58.8	13.1	965.1	583.1,1347.1
Girls (n = 333) ^c^					
115.0–119.9 (n = 3)	117.3	22.7	1.5	388.3	348.4, 428.3
120.0–124.9 (n = 5)	122.2	27.8	3.3	386.0	346.2, 425.8
125.0–129.9 (n = 16)	127.6	27.7	4.3	412.4	369.5, 455.3
130.0–134.9 (n = 47)	132.5	28.8	3.4	413.0	385.9, 440.1
135.0–139.9 (n = 57)	137.5	30.6	3.4	446.7	414.5, 478.8
140.0–144.9 (n = 64)	142.0	33.9	4.8	481.2	449.3, 513.1
145.0–149.9 (n = 54)	147.5	37.5	6.9	527.7	492.5, 562.9
150.0–154.9 (n = 45)	152.0	40.7	4.7	577.7	542.4, 613.1
155.0–159.9 (n = 23)	157.0	45.6	8.0	621.9	554.0, 689.8
160.0–164.9 (n = 18)	161.6	51.2	8.5	681.6	627.7, 735.6

^a^ Data presented are means.

^b^ There were no data between 115.0 cm and 124.9 cm.

^c^ The data for one girl (height>165.0 cm) were not considered.

**Table 4:** Ideal 24-h urinary creatinine excretion for height of 8–13-year-old Chinese children.

	Creatinine excretion (mg/d)	
Height (cm)	Boys	Girls
115		389.4
117		388.4
119		387.5
121		386.6
123		389.9
125		399.7
127	458.5	409.5
129	458.7	412.6
131	458.9	412.8
133	462.3	416.4
135	483.3	429.9
137	504.3	443.3
139	516.0	458.2
141	522.6	473.5
143	535.3	489.7
145	556.9	506.6
147	578.5	523.5
149	599.2	544.4
151	619.6	566.6
153	646.1	586.5
155	677.3	604.2
157	708.5	621.9
159	743.7	647.9
161	778.8	673.8
163	823.5	
165	882.5	
167	941.5	

The ideal 24-h urinary creatinine excretion for height is calculated by line interpolation of the mean creatinine excretion values from Table 4 for given 2-cm increments in mean height [5].

## Discussion

Urinary creatinine measurements are widely used in clinical and research to 1) access physical muscle mass, 2) examine the completeness of 24-h urine collections, and 3) serve as a parameter to adjust other urinary biological analytes in spot samples of urine. An abundance of knowledge is available on urinary creatinine reference values for adults [18–21], but hardly any for children have been reported in the past two decades. Urinary creatinine excretion varies markedly by anthropometric factors and by ethnicity [22, 23]. Therefore, the reference ranges for adults cannot be applied to children.

Many studies have reported data on the analyte to creatinine ratios in children [5, 24–28]. Age- and sex-specific 24-h creatinine reference values are necessary to determine analyte excretions from the analyte to creatinine ratios. For pubertal children, taking anthropometric variables into account is necessary while establishing urinary creatinine reference values. Age considerably affects body composition in growing children. Thus, 24-h urinary creatinine excretion reference ranges should be established according to age (e.g., 8–13 years). In addition, body height and weight are highly correlated with total muscle mass, and should, therefore, be adjusted for when establishing reference ranges for creatinine. Therefore, 24-h urinary creatinine excretion is normalized to individual body height and weight in establishing age- and sex-specific creatinine reference ranges. In the present study, the ratios of creatinine to body weight differed by sex. Our results are similar to those of Remer et al., who in their study on German children, found that the 24-h creatinine excretion of boys was 1.3 mg/kg/day higher than that of girls [5].

Urinary creatinine output for a given height is sometimes used to estimate lean body mass, although this estimate may be confounded by factors such as diet, vigorous exercise, infection, fever, and renal function. This method is based on the assumption that the amount of creatinine excreted reflects the muscle mass. Despite limitations, urinary creatinine excretion for a given height may be used as a convenient proxy for protein status [29, 30]. The ideal 24-h urinary creatinine for height is the standard value of daily creatinine excretion for a given height in an appropriately defined reference population. Using these reference values, the ratio of an individual’s 24-h creatinine excretion over the standard value for the same height can be used to assess the individual’s nutritional status. That is the creatinine height index (CHI), which is expressed as a percentage. $\text{CHI}\;\left(\%\right)=\frac{24-\text{h urinary creatinine excretion of subject}}{\text{ideal}\;24-\text{h urinary creatinine of same heifht}}\times 100$. The normal CHI is close to 1.0 in well-nourished children and ranges from 0.25 to 0.75 for protein-calorie malnourished children [29].

Data for ideal creatinine excretion for height derived from healthy Chinese children have previously been published. The only article to date establishing ideal creatinine excretion references for height was based on healthy white children aged 3–18 years [5]. In this study, 24-h urinary creatinine excretion was reported for a 90–186-cm height range. In the same paper, data from 9–13-year-olds were calculated as a single block. These values were uniformly higher than those presented in Table 4. Differences between the two studies may be explained by grouped age blocks employed and different dietary protein intakes. In our study, data from 9–13-year-olds were not merged due to differences in different age groups. The values for ideal 24-h creatinine excretion for height are the first for Chinese children based on a large-scale survey.

Our study has a few limitations. First, differences in genetics, muscularity, and amount of exercise in different populations might result in extrapolation biases. Second, because the accuracy of the adopted laboratory method may vary, the main finding should be extrapolated with prudence to other methods. Nevertheless, our findings are of great assistance to researchers in the same domain.

## Conclusions

To the best of our knowledge, this is the first large-scale epidemiologic study to include repeated 24-h urine collections to establish age- and sex-specific 24-h creatinine reference range for children. The anthropometry-based creatinine reference values for Chinese children be used to 1) identify the completeness of 24-h urine collections, 2) estimate average 24-h excretion rates of solutes from spot urine samples, and 3) assess the relative muscle mass of children.

## Supporting information

S1 Checklist (PDF)

S1 Dataset (XLSX)
